# Lima soundscape before confinement and during curfew. Airplane flights suppressions because of Peruvian lockdowna)

**DOI:** 10.1121/10.0002112

**Published:** 2020-10-05

**Authors:** Walter Montano, Elena Gushiken

**Affiliations:** 1 Technical Department, Arquicust, Gualeguaychu, Argentina; 2 Arquicust, Lima, Peru

## Abstract

Peru declared a state of emergency on March 16 in order to prevent SARS-CoV-2 (severe acute respiratory syndrome coronavirus 2) transmissions; thus, the International Airport was closed and the soundscape of urban zones under the flight tracks have been changed in view of the fact that airplane traffic was suspended. The authors have been conducting noise monitoring since February and because of that sufficient noise data for knowing the soundscape before and during the lockdown were obtained. This article presents a case of aircraft annoyance noise in one of Lima's city districts, which is near the aircraft climbing curve, toward the ocean on departure from Lima.

## I. SOUND LEVELS AND SOUNDSCAPE AT ONE OF LIMA'S CITY DISTRICTS

The office of ARQUICUST (a company dedicated to work in acoustics fields) is at Magdalena del Mar, one of Lima's city districts, and because the surrounding urbanization is under the flight pass of airplanes which take off from Jorge Chávez International Airport at 7.76 km (4.82 mi), it is important to monitor the environmental noise level in order to know the soundscape.

**FIG. 1.**
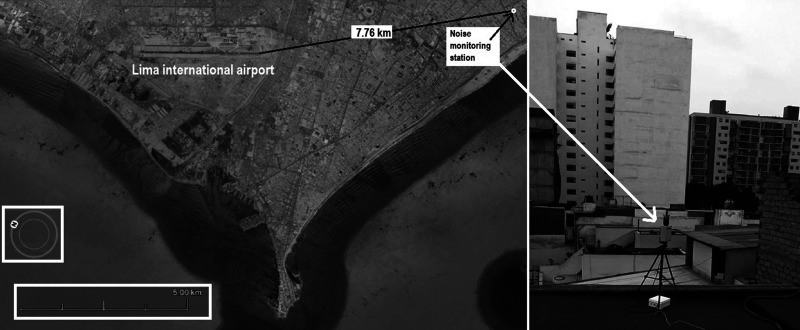
Picture of Lima's airport and the monitoring station location.

The monitoring station is a *TA120 noise sensor* manufactured by CESVA® (Barcelona, Spain), and the instrument comprises a class 1 sound level meter that records the sound level every second and transmits one average sample every minute to a NoisePlarform® cloud located in Barcelona (CESVA, 2020), therefore, it is possible to access the data from any place, and in this case, from Argentina. Figure 1 shows the relative location between both points and a picture of the monitoring station on the rooftop.

### A. Sound levels in Magdalena del Mar before Peruvian lockdown

The monitoring station is on a third-floor rooftop, and the apartment is in the middle of a block far away from any avenue (12°05′32.40″S–77°03′55.45″O). This location is important because the city background noise is low enough to measure the noise from passing airplanes flying east-south toward the ocean to leave Lima City.

**FIG. 2.**
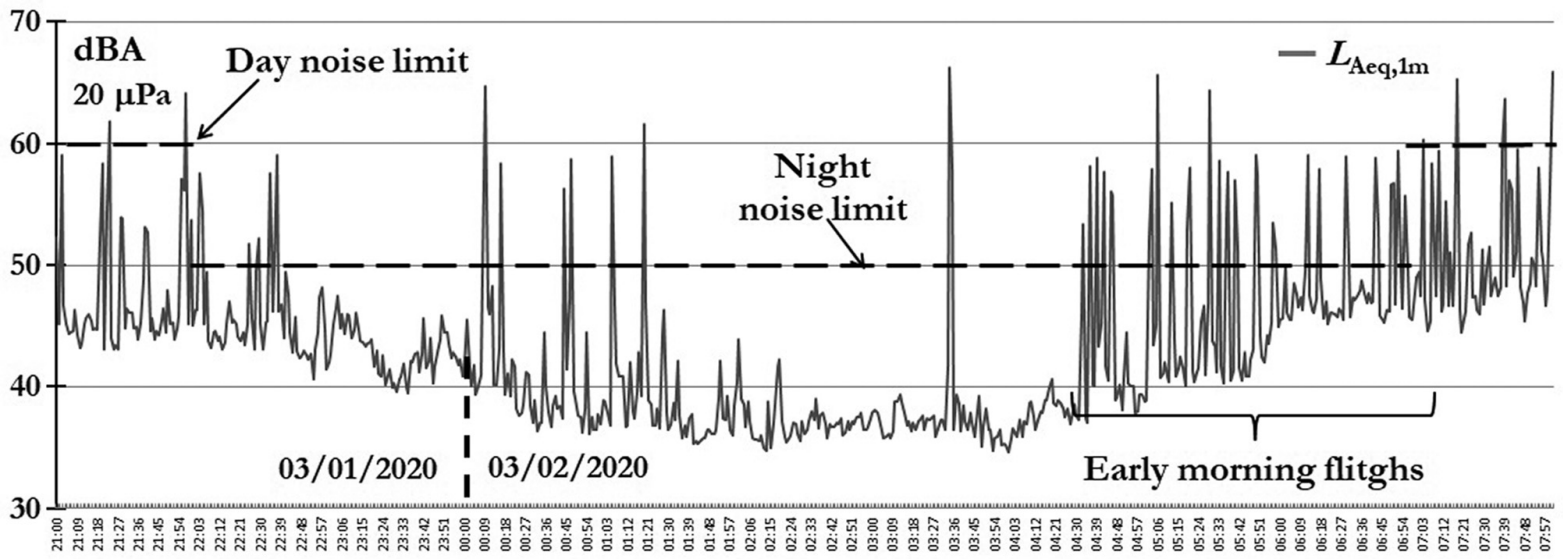
Night hours' time history between two days.

The airplane noise is annoying during night hours but is really disturbing at dawn because during the early morning hours, the flights from Lima to Peruvian provinces are numerous; although the numbers of passing flights are important, during day light hours, the airplanes' noise is masked by urban activities. Figure 2 shows the average one minute *A*-equivalent sound level (symbolized as *L*_Aeq,1min_) time history of one nighttime soundscape, where is possible to see the high noise levels because of airplanes passing by.

It is important to be clear that the noise sensor is intended to study the environmental sound levels, and it does not register the *L*_ASmax_—according to ISO 20906:2009—the most appropriate indicators for airplane noise measurements (ISO, 2020).

One goal of this article is to evaluate the real impact of aircraft noise on regular human activities and, basically, on sleeping during dawn hours. Therefore, the analysis was conducted since the end of February, before which was summer holiday time.

**FIG. 3.**
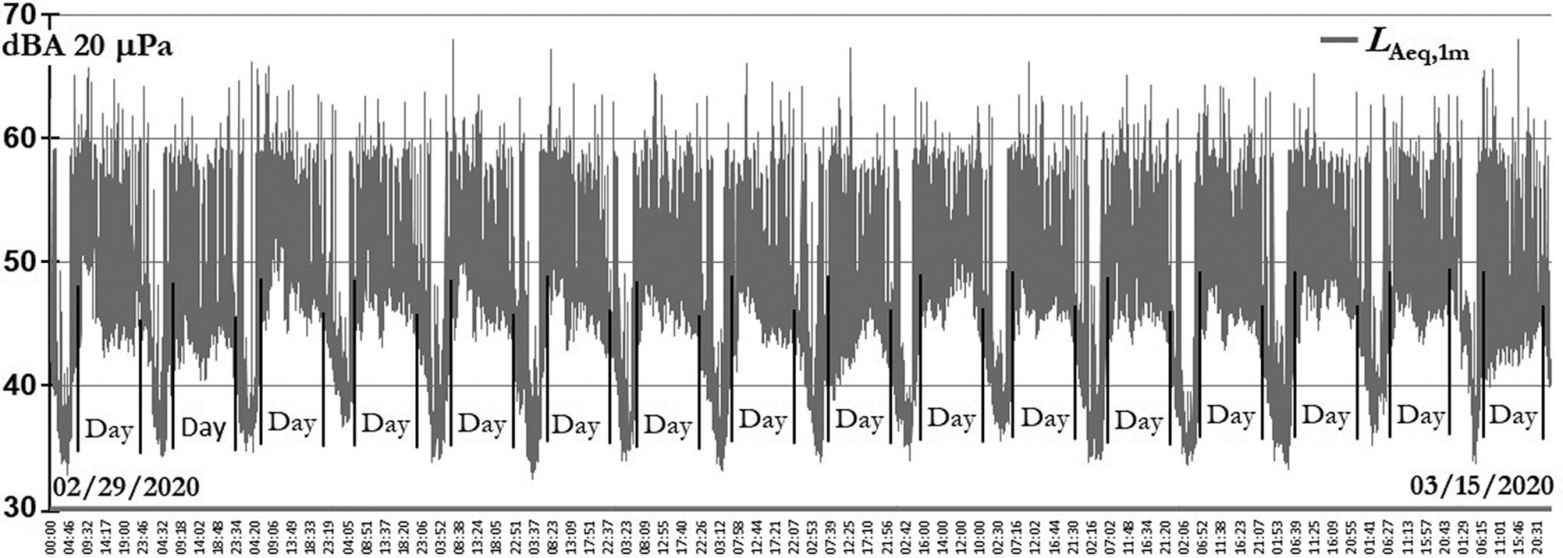
Magdalena del Mar sound levels before the lockdown order because of COVD-19.

Figure 3 shows the *L*_Aeq,1min_ time history from February 29 (00.00 h) to March 15 (23.59 h), March 16 was uncommon because of the lockdown; the daytime interval hour's—symbolized as “Day”—is also identified in the chart.

The NoisePlarform^®^ (located in Barcelona) facilitates the data analysis, does not require the installation of software since the entire service is cloud-based (Internet of Things or IoT), it uses a secure server certified with 256-bit SSL encryption, and can display project information in real-time; by using its several tools, it is possible to achieve precise, reliable, and secure calculations for equivalent sound levels and percentile levels.

For L_den_ calculation, the software uses the day-evening-night noise indicator in Eq. (1) as defined in ISO 1996-1 (2016), and in all cases, the units are dBA,

$$L_{\text{den}}=10\;\text{lg}\frac{1}{24}\;(12*10^{L_{\text{day}}/10}+4*\;10^{(L_{\text{evening}}+5)/10}+8*10^{(L_{\text{night}}+10)/10}).$$ (1)

The reference time for Peruvian legislation on environmental noise considers only day period (15 h) and night period (9 h; MINAM, 2003), but that is not used here. In order to make this article more communicable, ISO 1996-1 (2016) and the fact that the research in this matter was being conducted mostly by European countries (EU, 2002), the noise indicator *L*_den_ (*A*-weighted) was chosen with the following time intervals:

• *L*_Day,12h_ (or *L_D_*) is the continuous equivalent sound level from 06.00 to 18.00 h,
• *L*_Evening,4h_ (or *L_E_*) is the continuous equivalent sound level from 18.00 to 22.00 h,
• *L*_Night,8h_ (or *L_N_*) is the continuous equivalent sound level from 22.00 to 06.00 h.

### B. Sound levels in Magdalena del Mar after the “state of national emergency” order

The Peruvian government decreed the *state of national emergency* on March 16, which includes quarantine, confinement, social distancing, closing down the nonessential activities, and airport lockdown for commercial services after which only humanitarian flights were allowed. Because people have reacted in dissimilar ways to SARS-CoV-2 (severe acute respiratory syndrome coronavirus 2) outbreak, the President has imposed several stages to prevent social interactions in Lima.

#### 1. Stage 1: Confinement and social distancing

**FIG. 4.**
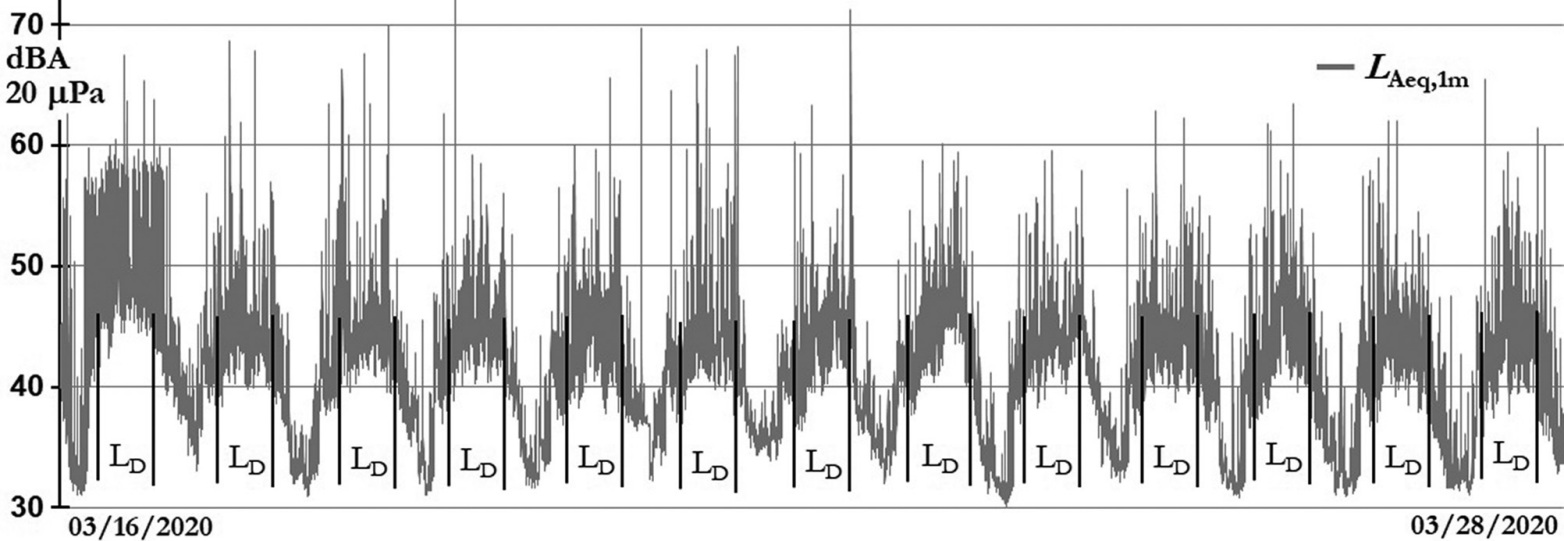
Magdalena del Mar sound levels during confinement and social distancing.

From March 16 to 28, the government order was to keep social distancing and to stay at home. Figure 4 shows the *L*_Aeq,1min_ evolution during those days; daytime interval hours are identified as *L*_D_.

The numbers of humanitarian airplane flights were slowly diminishing as one can see in Fig. 4.

#### 2. Stage 2: Confinement and a ban on circulation in the streets

People did not care about the COVID-19 propagation, so the president decreed a ban on circulation in the streets from March 29 to April 5. Due to no buses on the streets and no people after 18.00 h outsides their homes, the soundscape was very quiet to the people, and the sound levels reached a level lower than ever before.

**FIG. 5.**
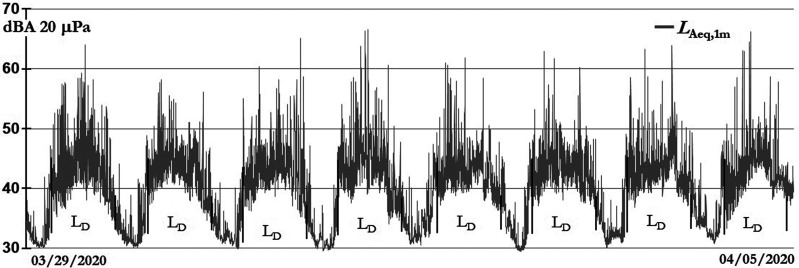
Magdalena del Mar sound levels during a ban on circulation in the streets.

Figure 5 shows the *L*_Aeq,1min_ evolution of these atypical days, only a few commercial flights were allowed, and it is possible to see how the sound levels reached their lowest values.

#### 3. Stage 3: Restricted social activities and night curfew

**FIG. 6.**
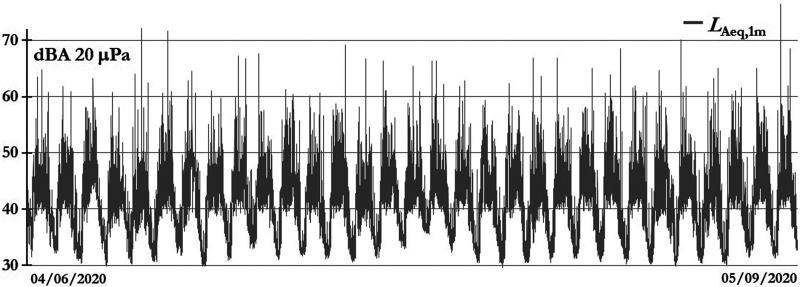
Magdalena del Mar sound levels under restricted social activities and night curfew.

The government had to make some concessions, and they have been permitting some social activities, such as going to shop at the supermarket Monday–Saturday, but some restrictions were applied, such as a curfew after 18.00 h and a ban on Sunday social activities. Figure 6 shows the *L*_Aeq,1min_ evolution from April 6 to May 9, in which the maximum levels correspond to some authorized flights (people who had to return to their countries or entry to Peru) and humanitarian flights.

**FIG. 7.**
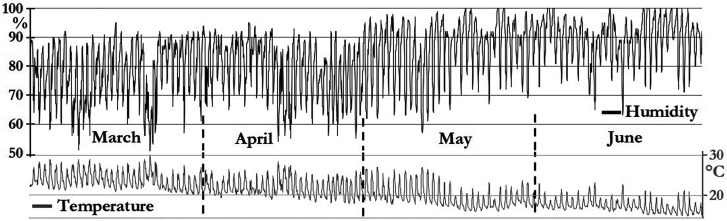
Magdalena del Mar weather conditions from March 1 to June 30.

The interval time up to May 9 was chosen arbitrarily because in those autumn days, the temperature was going down and the humidity was going up (see Fig. 7), so people stayed at their homes more and, consequently, the noise generated by this “new social interaction” modified the sound levels. But, its analysis is not part of this article, and the goal is to characterize the airplane noise.

In Fig. 7, the left axis represents the percentage of relative humidity (black line); the right axis represents the temperature in Celsius degrees (grey line). The atmospheric data were taken from “*Campo de Marte*” weather station (SENAMHI, 2020) at 3.5 km from the monitoring station.

#### 4. Stage 4: The last confinement week before the opening on July 1

**FIG. 8.**
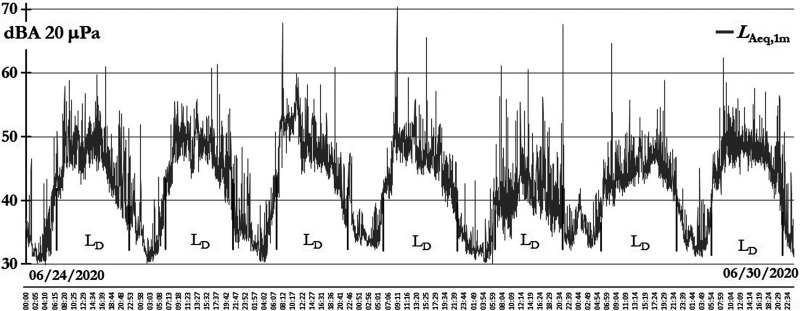
Magdalena del Mar, the last confinement week sound levels before opening on July 1.

On July 1, after 107 days of confinement due to COVID-19, the Peruvian government announced the end of confinement in Lima and other Peruvian regions. In Fig. 8, the *L*_Aeq,1min_ evolution of these days is shown; daytime interval hours are identified as *L_D_*.

In Fig. 8, is possible to see that nighttime sound levels reach 30 dBA, and some humanitarian flights occurred only in daytime and a few occurred during night hours. The last week before the opening, from June 24 to June 30, a “new normal” is presented in order to analyze an atypical sound level condition. There was much movement by the people because the government authorized more commercial activities and some industrial factories opened and construction activities were allowed.

## II. SOUND LEVELS ANALYSIS BEFORE/DURING STATE OF EMERGENCY

In order to understand the sound level changes in this Lima district due to COVID-19 confinement, Table I summarizes all sound levels of each stage scenario for each interval time.

**TABLE I.** Summary of *L*_den_ noise indicator for each stage scenario (dB re 20 μPa).

Stage	*L_D_*	*L_E_*	*L_N_*	*L*_den_
Before lockdown	52.0	51.0	47.7	55.3
Confinement and social distancing	47.5	47.7	39.7	49.6
Confinement and a ban on circulation in the streets	46.7	44.3	35.6	47.0
Restricted social activities and night curfew	47.2	44.8	36.6	47.6
The last confinement week	48.8	45.7	36.5	48.6

In Table I, it is possible to see some important issues:

• During the *confinement and social distancing*, the night noise level or *L_N_* was 8 dBA less than before the lockdown.
• The lowest night noise levels were observed during *confinement and a ban on circulation in the streets*, and during that week, the night soundscape was almost 12 dBA less than before the lockdown.
• Sound levels in the last confinement week show +2.1 dBA during daytime as a result of people's activities; a +1 dBA during the nighttime probably is caused by distant noise sources as the high humidity does not much affect the sound waves' propagation.

### A. Sound level analysis by means of statistical tools

**FIG. 9.**
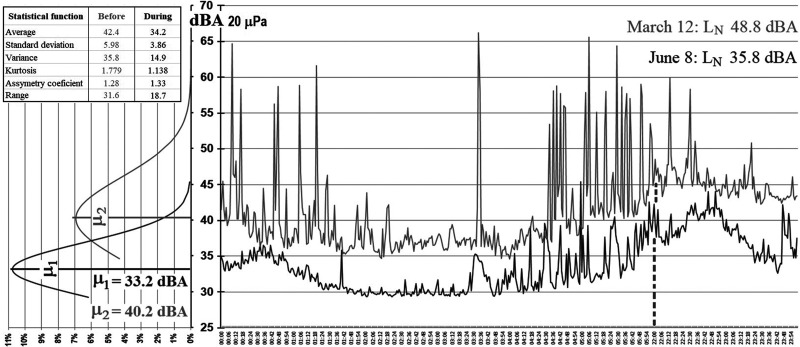
Normal distribution of two *L*_N_ sound levels.

Among the statistical analyses that could be performed, the normal distribution is one of the simplest to understand. Figure 9 shows the night sound level evolution of two different Mondays and their normal distribution: before lockdown (grey line) and during confinement (black line). It is interesting to analyze the special case of environmental sound level normal distribution because the values have a discrete dispersion, and due to the absence of sound levels below 34 dBA (for March 12) and 29 dBA (for June 8th), they have a truncated distribution.

The normal distribution for March 12 (the noisier Monday before lockdown) has a mesokurtic behavior with a median value of 40.2 dBA, meaning that the sound levels are distributed above 40.2 dBA; and for June 8 (the less noisy Monday during confinement), the distribution has a leptokurtic behavior with a median value of 33.2 dBA, meaning that the sound levels are concentrated around 33.2 dBA. The soundscape perception during confinement is quieter than before.

### B. Sound level behavior analysis by means of percentile sound levels

In order not to confuse the reader with one specific ISO term, and because the same idea is used worldwide, the authors will refer to the *percentile levels* according to ANSI/ASA S12.100 (ANSI/ASA, 2014) instead of “percentage of exceedance” (ISO, 2017). Table II summarizes the *L_D_*, *L_E_*, *L_N_* (just for one week as an example), and the *L*_10_, *L*_90_ for each day-evening-night individually.

**TABLE II.** Day-evening-night sound levels and their percentiles: March 1–7 (dB re 20 μPa).

Day	*L_D_*	*L*_10,__*D*_	*L*_90,__*D*_	*L_E_*	*L*_10,__*E*_	*L*_90,__*E*_	*L_N_*	*L*_10,__*N*_	*L*_90,__*N*_
March 1	50.8	56.2	42.4	51	54.4	44.2	47.1	47.6	38.4
March 2	53.7	57.4	46.6	51.3	54.6	45.9	49.1	47.3	39.5
March 3	52.2	56.4	46.1	51	53.8	45.2	48.3	47.3	40.4
March 4	52.3	56.2	45.6	50.5	54.0	45.1	49.6	49.2	39.2
March 5	53.1	56.9	46.0	50.6	54.2	44.1	47.4	45.5	38.4
March 6	52.2	55.5	46.6	51.2	54.9	44.9	47.6	47.5	38.3
March 7	51.6	55.8	44.8	50.6	53.7	44.0	47.4	48.4	39.3

**FIG. 10.**
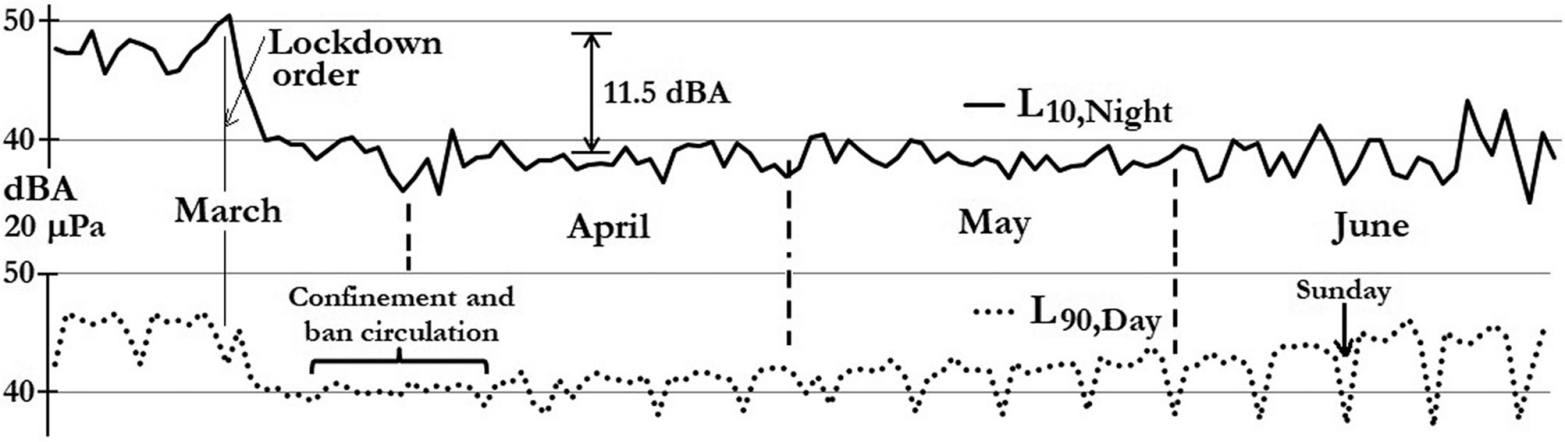
Percentile evolution from March 1 to June 30: *L*_A10,Night_ (solid) and *L*_A90,Day_ (dotted).

The act of analyzing the sound levels by *L*_den_ allows acousticians to have three values for each day, so it is interesting to use the percentile levels of each period to improve the examination. Due to the sound levels high variance (σ^2^), the authors have used the percentile levels evolution in order to observe the smooth fluctuation of the sound levels for each period; the use of percentile level is proposed in a recent investigation in order to establish a taxonomy *for the assessment of the changes in soundscape resulting from the COVID-19 lockdown* (Asensio *et al.*, 2020). Figure 10 shows only the *L*_10,Night_ (10th percentile for night hours) as a solid line, and the *L*_90,Day_ (90th percentile for day hours) evolutions as a dotted line. In a comparison between *L*_10,Night_ and *L*_90,Day_, it is interesting to see that the *L*_90,Day_ evolution gives a better idea of how the “background” sound levels are increasing gradually because of the anthropogenic noise produced by people's movement due to de-escalation.

On the other hand, the *L*_10,Night_ evolution is better for showing the high impact of airplane noise before the lockdown and how the night sound levels dropped 11.5 dBA because there are no flights at dawn. Another interesting thing to note is how the Sunday's *L*_90,Day_ level dives down, and this was because on Sundays, street circulation was forbidden.

## III. CONCLUSION

Acousticians have been able to register a distinct soundscape during the lockdown due to SARS-CoV-2 at the end of which it will be important to communicate to the authorities this information on healthy environmental sound levels. This will enable them to improve governmental actions in order to keep the environmental noise as low as is possible. The authors have a lot of data to process, and they are writing a computer program (Montano, 2018) to eliminate outliers. A special scenario took place: It was possible to hear at every dawn a birds' call concerto, and the neighborhood with no early flights could wake up in a healthier mental state.
